# Determination of the Operational Parameters for the Manufacturing of Spherical PVP Particles via Electrospray

**DOI:** 10.3390/polym13040529

**Published:** 2021-02-10

**Authors:** Christian Narváez-Muñoz, Pavel Ryzhakov, Jordi Pons-Prats

**Affiliations:** 1 Escola Tècnica Superior d’Enginyers de Camins, Canals i Ports, C/Jordi Girona 1, Campus Nord UPC, Universitat Politècnica de Catalunya—BarcelonaTech (UPC), 08034 Barcelona, Spain; pryzhakov@cimne.upc.edu (P.R.); jpons@cimne.upc.edu (J.P.-P.); 2 Centre Internacional de Mètodes Numérics en Enginyeria (CIMNE), C/Gran Capitán s/n, Campus Nord UPC, 08034 Barcelona, Spain

**Keywords:** electrohydrodynamic atomization, Taylor cone, stability island, scaling law, numerical simulation, experimental, viscosity

## Abstract

This work aims at bridging experimental and numerical approaches to determine the optimal operating parameters for the fabrication of well-shaped polyvinylpyrrolidone (PVP) particles via electrohydrodynamic atomization. Particular emphasis is given to the role of the PVP solution viscosity. Solutions of PVP at various concentrations dissolved in Dimethylformamide (DMF) were prepared and analyzed. Numerical simulation using a coupled electro-CFD model was used to determine the ranges of experimental flow rate and the voltage, ensuring that well-shaped spherical particles are produced. It was deduced that the optimal combination of the parameters (flow rate, voltage, and polymer concentration) can be well approximated by a scaling law. The established relationship allowed determination of a stability island that guarantees that the given polymer solution will form spherical particles. Analyzing morphology and sizes of the particles manufactured in the optimal parameters range, we show, among others, that the size of the PVP particles can be predicted as a function of the flow rate by a power scaling relationship.

## 1. Introduction

**Figure 1:**
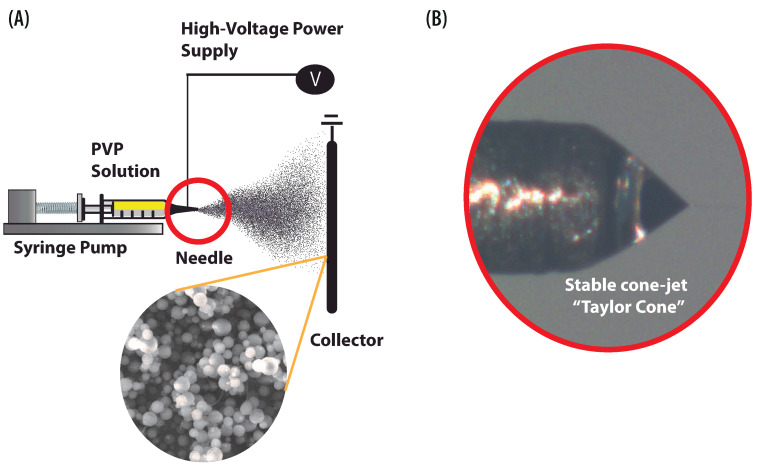
EHDA process for manufacturing particles. (**A**) Schematic configuration and main elements of the electrospray setup. (**B**) Illustration of the stable conical shape “Taylor Cone” during the manufacturing of polymer particles.

Electrohydrodynamics (EHD) is the study of the interaction between electric fields and electrically charged fluids. EHD phenomena are used in various practical applications. These include advanced mass spectrometry, EHD pumps, manufacturing and EHD thrusted. Substantially important EHD applications are the manufacturing techniques based on the electrohydrodynamic atomization (EHDA) also known as “electrospray”. Although EHDA can be used in conjunction with various materials, we shall concentrate in the present paper on the electrospray applied to polymer solutions. During the manufacturing of polymer structures by electrospray, the solution is pumped out at a constant flow rate. Meanwhile, a high voltage is applied between the polymer solutions and collector (Figure 1A). The electric and hydrodynamic forces put the solution into motion expelling it from the end of the needle forming a cone-jet, which breaks up into droplets due to axisymmetric instability [1]. The charged droplets split into multiple droplets to evaporate the solvent. Ultimately, droplets solidify generating submicrometric particles.

### 1.1. Motivation

Even though electrospray has been known for more than a century, only during the last two decades it started being successfully used for manufacturing micro to nano-size structures. These kinds of structures are particularly attractive for pharmaceutical and bioengineering applications [2]. Different polymers can be processed to promote electrosprayed structures with different shapes [3]. For example, Polyvinylpyrrolidone (PVP) is one of the most widely used polymers, which is featured by its versatile properties being biocompatible, non-toxic, bioactive, water-soluble, and biodegradable. These properties triggered a quick development of applications in drug delivery, as well as encapsulation by using spherical particles.

Among other benefits, spherical shape has the ability of overcoming the high interstitial pressure and improve the efficacy of vascular tumor therapy [4]. Novel investigations demonstrate that polymeric spheres lodge in damaged tissue whereas particles with other shapes tend to accumulate in healthy tissue [5,6]. The spherical shape of the particles can be obtained only in case of adequately choosing EHDA operating parameters and the material properties of the charged fluid. Unless these parameters are appropriately chosen, the deformed morphology (stretched particles or even fibers) is obtained. Consequently, considerable attention is focused on developing and predicting the correct operating parameters to produce spherical particles with high performance and at low cost.

### 1.2. EHDA and Modes

The first theoretical description of this phenomenon was given by Taylor [7], who calculated the semi-angle for an ideal case, considering that the electrostatic and hydrodynamic forces are in equilibrium. The conical shape of the meniscus formed during the electrospray process at a stable state, shown in Figure 1B, is known as Taylor’s cone. The shape of this cone is quantified by its associated half-angle and critically depends on the physical properties of the solutions, such as; conductivity, surface tension, and viscosity. For a solution of water mixed with oil, Taylor [7] estimated the half-angle as 49.3°. Taylor’s model has motivated fundamental research, which resulted in establishing the scaling laws to approximate the diameter of droplets and jet, the onset operating parameters (minimum flow rate $Q_{o}$, electric potential $\phi_{o}$), and the total emitted current [8,9,10,11,12].

**Figure 2:**
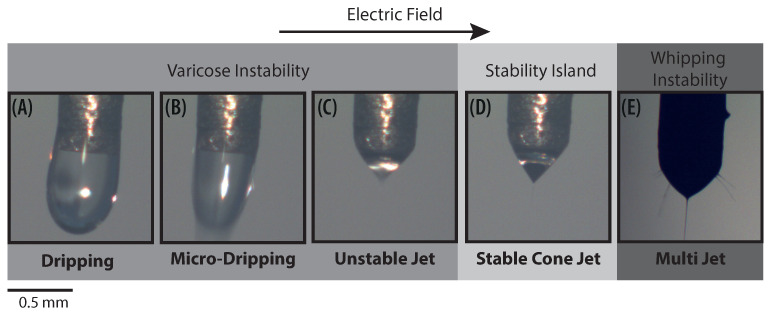
Photographs of the main operation modes of the Electrospray technique for a concentration of 13% PVP and a flow rate of 0.1 mL/h. (**A**) Dripping 4.9 kV, (**B**) Microdripping 5.5 kV, (**C**) Unstable Jet 6.3 kV, (**D**) Stable Cone-Jet 8.5 kV, and (**E**) Multi-Jet 11.2 kV.

Cloupeau et al. reported that for a set of given operating parameters the cone changes across different *operation modes* or *regimes* as the applied voltage increases [13]. In addition to the operating parameters, the stability of the cone also depends on ambient conditions and geometrical features [14]. In Figure 2, the shape of the meniscus for the five principal operation modes can be observed. These modes have been photographed by varying the applied voltage for a fixed PVP concentration of 13% and a flow rate of 0.1 mL/h. A semispherical meniscus is formed at the end of the capillary tube and nearly uniform drops are ejected from it. If the voltage increases further, the size of the drops reduces, ranging from ∼0.1 to ∼0.01 micrometers [15]. These two aforementioned modes are usually referred as *dripping* and *microdripping*, respectively (Figure 2). By increasing the voltage further, the semispherical meniscus stretches into a conical shape due to the electrostatic forces acting on the ions of the liquid. At this point, a thin jet is formed, and small droplets are emitted. Then the stable cone-jet is reached (Figure 2D). Upon further increase of voltage, fluid enters the so-called multi-jet mode (Figure 2E). It should be noted that the stable cone-jet destabilizes downstream in varicose or whipping disturbances. Several reports have shown that cone-jet is the most important spraying mode [1,16,17,18]. In particular, most of the applications aim to operate in the stable cone-jet regime, because it is the most reliable mode for manufacturing homogeneous particles. This mode generally operates in an area called the “stability island”, which corresponds to a narrow range of voltages and flow rates. Although cone-jet mode has been investigated previously, the stability map of polymer solutions at different concentrations is rarely addressed and requires further study and discussion.

As already mentioned, the cone-jet regime can be recognized by the formation of the Taylor’s cone. However, the values of the corresponding voltage and flow rate are not known a priori. Although the minimum values of these parameters can be approximated by scaling laws, it must be noted that liquid properties, such as viscosity, can limit their straight-forward application [19]. In fact, the minimum flow rate is altered by viscosity [20]. Moreover, the data from Munir et al. [21] suggests that the final morphology (i.e., formation of fibers or particles) as well as the final size of the structures are also affected by the viscosity (These limitation related to the viscosity could be overcome by adding supercritical CO_2_ (SC-CO_2_) to the polymer solution [22]. An innovative study performed by Baldino et al. [23], suggests that an electrospray process assisted by SC-CO_2_ reduces viscosity and surface tension of the solution to be processed and increases the production rate). Despite the influence noted above, studies on the effects of viscosity in the operating parameters to form a stable cone-jet are scarce. Present study focuses, in particular, on the role of the viscosity on the onset voltage.

### 1.3. CFD Studies

Efforts in theoretical and experimental studies of EHDA mostly focus on finding the optimal operating parameters [8,9,10,11]. However, to-date these procedures require using expensive resources and are time-consuming. To reduce the cost of EHDA studies, computational fluid dynamics (CFD) appears to be a viable option. Feng and Scott performed one of the first numerical simulations of a liquid exposed to an electric field using the Finite Element Methods (FEM) [24]. In this study, small deformations of a drop suspended in an immiscible fluid were simulated. Subsequently, Hartman et al. were able to simulate the Taylor cone and jet formation of various liquids with electric conductivity less than 13 μS/m, using a 1D model [25]. However, the results strongly differed from the experimental data. In the last few years, much more information on the liquids influenced by an external electric field has become available. Ghasemi et al. reported three-dimensional deformation of a drop under the effect of the electric field, using Direct Numerical Simulation (DNS) [26].

Lim et al. studied the formation of dichloromethane (DCM) droplets [27]. The model described the two-phase system using a single set of Navier–Stokes equations. Almost simultaneously, Wei et al. [28] and Xu et al. [29] used the same model as Lim et al., but with a different purpose. Wei studied the jet breakup and droplet formation of heptane [28]. In the recent study of Jiang et al. the prediction of the behavior of the cone-jet was improved by considering the effects of the space charge, the relative error between the experimental data and the numerical simulation of ethanol cone length decreased from 12.5% to 4.2% by including the effect of space charge in the model [30]. The outcomes of the mentioned studies confirm that the CFD is a viable option for approximating the temporal and spatial evolution of cone-jet by varying the voltage and flow rate. This study intends to employ the numerical simulation to elucidate the optimal operating parameters that allow suppressing of downstream disturbances to find the stability island.

In the present study, we strive to bridge the experimental and the numerical approaches to establish a strategy for manufacturing well-shaped spherical PVP particles. We first characterize several PVP solutions experimentally and then perform numerical simulation using the obtained properties and experimental data. The aim consists of identifying electrospray operation parameters that lead to stable cone-jet regime and, thus, are most favorable for manufacturing spherical particles. Afterwards, using the numerical simulation results we deduce a scaling low relating the process parameters. This data is ultimately used in the last experimental step when the PVP particles are actually manufactured. We highlight the impact of three parameters, namely the PVP concentration in the solution, flow rate, and electric field, upon the final shape of the electrosprayed structure.

## 2. Numerical Model: Two-Phase Solver with an Electric Coupling

### 2.1. Governing Equations for the Fluid Flow

In the following, we describe the numerical model implemented in the present work. The simulation of electrospray of the PVP solution requires considering the liquid phase (PVP solution) and the gas phase (air). The behavior of the two phases involved is governed by the continuity equation and conservation laws. Assuming that the two phases are incompressible, the continuity equation can be expressed as:

(1) ∇·U=0

The momentum equation for the fluids can be written as:

(2) $$\frac{\partial \rho U}{\partial t}+\rho U\cdot \nabla U=-\nabla p+\nabla \cdot \left(\tau_{s}+\tau_{p}\right)+\nabla \cdot \tau_{e}+\rho g+F_{st}$$

where *p* is the pressure, **U** is the velocity, *g* is the gravity and ρ is the density. The surface tension force term $F_{st}$ (which must be added because in sub-millimeter droplets produced in EHDA surface tension force is not negligible) is expressed by γ, κ and $\delta_{s}$, which are the surface tension coefficient. The viscous stress tensor is composed by Newtonian ($\tau_{s}$) and viscoelastic $\left(\tau_{p}\right)$ contribution. Usually in the electrospray process liquids show Newtonian behavior [16,31,32,33,34]. Therefore, the viscous stress tensor reduces to $\nabla \cdot (\tau_{s})$:

(3) $$\tau_{s}=[\mu \left(\nabla U+\nabla U^{T}\right)$$

where μ is the viscosity of the fluid. To model the impact of the electric field upon the fluid flow, the force term of the momentum equation must include the electric stress, which is the divergence of the Maxwell stress tensor $\tau_{e}$.

For capturing the evolution of the liquid–gas interface Volume-of-Fluid (VoF) approach of Hirt and Nichols [35] is used here. Using alternative schemes, such as Level Set [36,37,38] or moving grid Lagrangian approaches [39,40,41] is also possible; however, their use for the problem at hand may be complicated due to the expected topology changes in the liquid domain.

VoF method consists of solving the phase fraction equation:

(4) $$\frac{\delta \alpha }{\delta t}+\nabla \cdot \left(\alpha U\right)=0$$

The phase fraction α can take the following values: 0 for outer phase (gas in our case), 1 inner phase (liquid in our case) and 0 <α< 1 liquid–gas interface. The interFoam solver of the 2.3.1 OpenFOAM software used in the present work can model the two-phase fluid flow. We added the electrohydrodynamic coupling to the mentioned solver. This coupling, as already mentioned, consists of adding the divergence of the Maxwell stress tensor to the residual of the momentum equation. In the following subsection, we describe the computation of Maxwell stress $\tau_{e}$, considering the specific features of the problem at hand. The inclusion of this term in the momentum equation defines the electrohydrodynamics coupling of the model.

### 2.2. Maxwell Stress Tensor

The Maxwell stress tensor represents the electrostatic force, consequently, it is essential for modeling EHD problems. According to Chen, there are two approaches to derive the electrical force density $\nabla \cdot \tau_{e}$, namely Kelvin and Korteweg–Helmholtz ones [42]. The Kelvin force density (Equation (5)) accounts for the microscopic electromechanics effects. Melcher suggested that this force is the sum of the Coulombic and polarization forces [43].

(5) $$F_{K}^{e}=\rho_{e}E+P\cdot \nabla E$$

where **E** is the electric field, and P is the polarization force, for the liquid dielectric $P=(\epsilon -\epsilon_{0})E$. The Korteweg–Helmholtz force density (Equation (6)) is deduced from the energy conservation principle, which is essential for the coupling the micro and the nano-fields. This force density is useful to predict the consequences of electromechanical coupling [44].

(6) $$F_{K-H}^{e}=\rho_{e}E-\frac{1}{2}E^{2}\nabla \epsilon +\nabla \left[\frac{1}{2}\rho {\left(\frac{\delta \epsilon }{\delta \rho }\right)}_{T}E^{2}\right]$$

The first term represents the Coulombic force (Equation (7)), the force that drives the electrohydrodynamic flow.

(7) $$F_{e1}=\rho_{e}E$$

Coulombic force is present in all the simplifications that we consider here. This force is perpendicular to the surface. The second term in the equation represents the permittivity gradient force (Equation (8)), which is constant for each liquid and is perpendicular to the surface.

(8) $$F_{e2}=-\frac{1}{2}E^{2}\nabla \epsilon$$

The third term represents the electrostriction force. Additionally, Melcher suggested that the effect, of the electrostriction force is absorbed by the hydrostatic pressure and therefore can be ignored [43]. Thus, in our case, the Korteweg–Helmholtz force density reduces to:

(9) $$F_{e}=\rho_{e}E-\frac{1}{2}E^{2}\nabla \epsilon$$

### 2.3. Governing Equations for the Electric Field

For Maxwell’s equations electroquasistatic approximation can be considered since the polymer solutions have low conductivity and the electrostatic process is dominant. In addition to this, Hua and Wang proposed to neglect the magnetic effects when the dynamic currents are small (note that liquids such as molten metals or semiconductors with a highly conductive can generate magnetic fields) [45]. In other words, the electric field can be assumed irrotational and expressed as:

(10) ∇×E=0

Since the magnetic effects are neglected, the electric field can be described in function of the applied voltage ϕ.

(11) E=−∇ϕ

Applying the Gauss’s law for an electrical linear medium, it can be reduced to:

(12) $$\nabla \cdot \left(\epsilon E\right)=-\rho_{e}$$

where $\rho_{e}$ is the free charge density and ϵ is the permittivity or dielectric constant. The free charge density is a function of the current **i**, described by the charge conservation equation (Equation (13)).

(13) $$\frac{\delta \rho_{e}}{\delta t}+\nabla \cdot i=0$$

The Ohmic model provides an excellent approximation, called leaky dielectric, which determines the interface charge density [46]. The Equation (13) reduces to the charge conservation equation in the Ohmic regime:

(14) $$\frac{\delta \rho_{e}}{\delta t}+\nabla \cdot \left(\rho_{e}U\right)=\nabla \cdot \left(\sigma \nabla \phi \right)$$

Once the charge distribution and the electric field are determined, then the electric stress can be calculated. However, for high conductive liquids with rapid charge relaxation, Chen [42] suggested that the governing Equation (14) is simplified to:

(15) ∇·σ∇ϕ=0

The velocity of charge relaxation is related to the electric conductivity and permittivity. Such physical properties are characterized by the following dimensionless numbers: electrical relaxation time (Equation (16)) and hydrodynamic characteristic time (Equation (17)). In the literature, $T_{e}$ is used to identify fluids with fast charge relaxation (typically $T_{e}<10$ s).

(16) $$T_{e}=\frac{\epsilon }{\sigma }$$

(17) $$T_{h}=\sqrt{\frac{\rho d_{o}^{3}}{\gamma }}$$

where σ, and γ are electrical conductivity, and surface tension of the polymer solution, respectively. *D* is the characteristic length (outer diameter of the needle).

**Figure 3:**
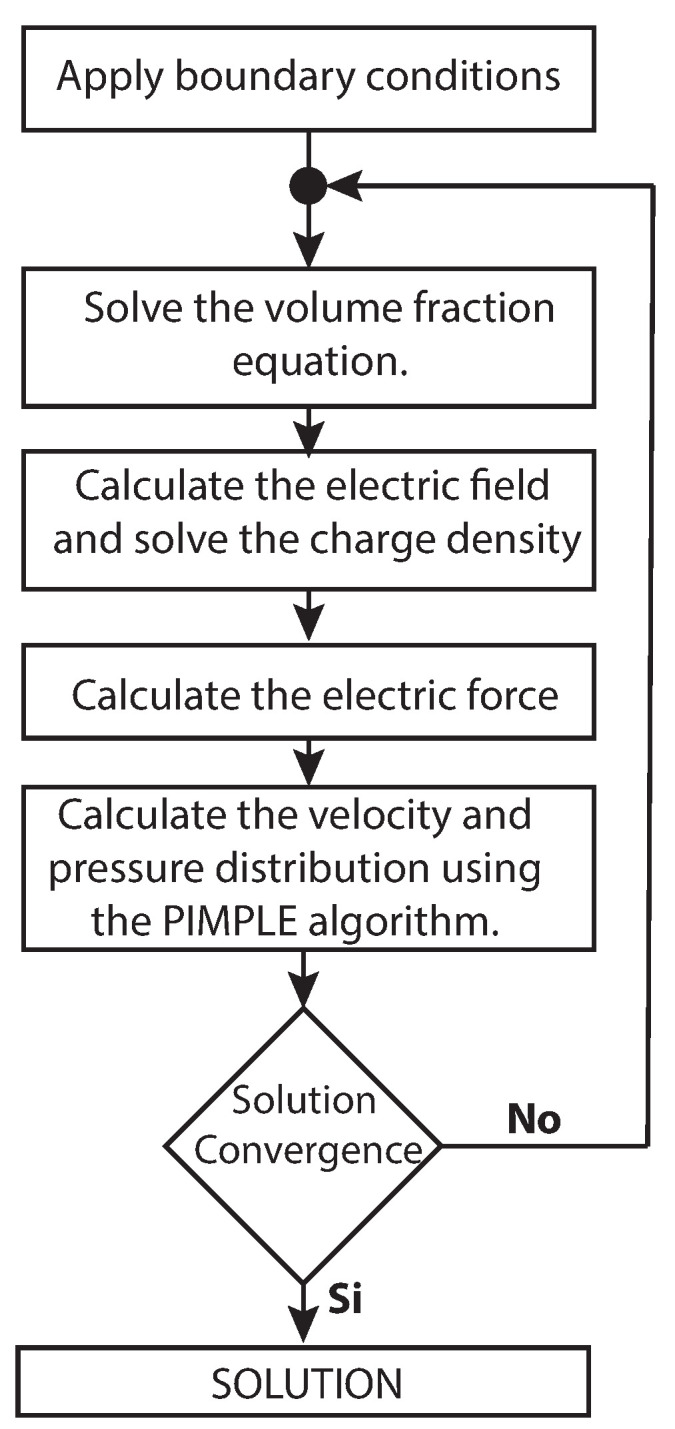
Algorithm employed for solving the EHDA problem.

To this end the different ingredients of the numerical model used here are presented. The over solution strategy is summarized in the flowchart shown in Figure 3. At the beginning of each time step, the volume fraction equation is solved to capture the liquid–gas interface position. Next, Equations (11) and (14) are solved to calculate the electric force (Equation (9)). Next, the Navier–Stokes equation (Equations (1) and (2)) is computed to determine the velocity and pressure fields in both fluids. The Navier–Stokes equations are solved using the so-called “PIMPLE” algorithm of the 2.3.1 OpenFOAM software.

## 3. Experimental Method

### 3.1. Material Characterization: Viscosity and Surface Tension Measurement

In this section, the experimental equipment and employed methodology are described. First, we describe the tools used for determining the properties of the solutions considered in the present work. For preparing the polymer solution, PVP K30 (molecular weight 27,000∼32,400 g/mol) purchased from Yuking (Shanghai, China), was dissolved in DMF (Fisher Scientific, Bogota, Colombia). The solutions of PVP in DMF were freshly prepared and stirred for 40 min at room temperature before each experiment. Various concentrations of PVP blended with DMF prepared in the present work are listed in Table 1.

**Figure 4:**
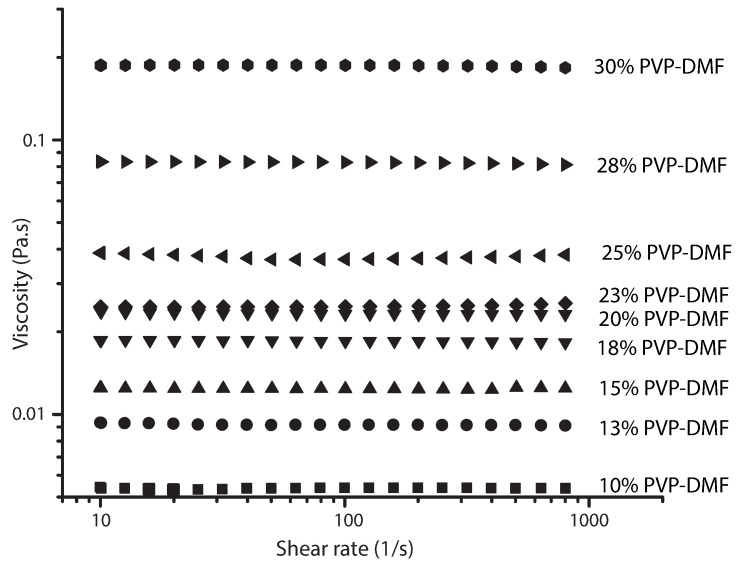
Viscosity vs. shear rate plots for PVP solutions in DMF.

The viscosity of the PVP solutions was measured by employing a rotational rheometer Discovery HR-2 from TA Instruments and geometry (concentric cylinders). A steady flow sweep was applied to each prepared PVP solution at shear rates ranging from 0.01 to 800 s${}^{-1}$, the PVP diluted solutions showed a constant viscosity during the shear interval (Figure 4). Therefore, the polymer solutions will be considered Newtonian fluids. Static surface tension was measured by an automatic digital tensiometer DyneMaster DY-300 (Kyowa Interface Science Co., Ltd., Saitama, Japan) using Wilhelmy Plate method [47]. The measurements were carried out at ambient conditions, each measurement was repeated three times and the average was taken.

### 3.2. Experimental EHDA Setup

For performing electrospraying the PVP solution was inserted into a 5 mL plastic syringe (NIPRO, Quito, Ecuador), and the flow rate of the polymer solution was controlled by a syringe pump (NE-300), the solution was pumped through a capillary tube (needle) of 0.4 mm of internal diameter. To produce the electric field, the tube was connected to a high voltage source (Genvolt, model 73030, 30 kV) and the collector was grounded. An analog CCD camera (DFK, model 22BUCO3) and IC capturing software was used to record Taylor cone and cone-jet formation. Sprayed PVP structures were deposited on the collector which was located at a distance of 12 cm from the capillary tube. This distance must ensure evaporation of the solvent prior to the deposition, if the solvent is not completely evaporated, the particles obtain a flattened shape. The equipment described above composes the basic electrospray setup, shown in Figure 1A. Used configuration allows easy modification of the voltage, flow rate, and the distance between the collector and the needle. To estimate the critical voltage at which spraying initiated, the flow rate was fixed to 0.1 mL/h. Then, voltage was varied until the PVP solution formed a cone. The experiments were performed varying voltage from 7.2 to 12 kV. Collection time of the electrosprayed particles was varied from 30 s to 5 min. All experiments were carried out at ambient conditions 21 ± 2 °C and relative humidity 48 ± 5%.

### 3.3. Characterization the Morphology of the PVP Structures

The scanning electron microscope (FEG-SEM, Mira3 Tescan) was used to characterize the size and morphology of the PVP structures. To observe the structures under the FEG-SEM the operation voltage was set between 3 to 5 kV. A sputtering evaporator (Quorum Q150 ES) covered the PVP structures samples with a conducting gold layer of approximately 20 nm. Micrographs using low magnification (×2000) were obtained to observe the size distribution. High magnification (×10,000) of micrographs was used to obtain the details of the morphology of the particles.

## 4. Numerical Simulations

The aim of the numerical simulation performed in the present work and described below is to identify the operating parameters that ensure a stable cone-jet regime for different PVP solutions. We perform the numerical simulations using the electromechanical two-phase solver described in Section 2 using PVP solutions properties specified in Section 3.1. In the present section, we first describe the simulation domain geometry (Section 4.1) and then proceed with the description of the simulation cases. The first simulation (Section 4.2) is performed using the operation parameters (flow rate and voltage) that are known to be leading to Taylor cone formation. This test is performed to test the numerical solver and ensure its applicability to the problem at hand. The second simulation (Section 4.3) is performed varying the operation parameters with the aim of identifying the ranges of flow rates and voltages that result in the stable cone-jet regime. Based on these simulations, the stable zone graphs are obtained. We use this data for establishing the stability islands and, consequently, for performing the fabrication experiments described in Section 5.

### 4.1. Geometry Domain and Boundary Conditions

**Figure 5:**
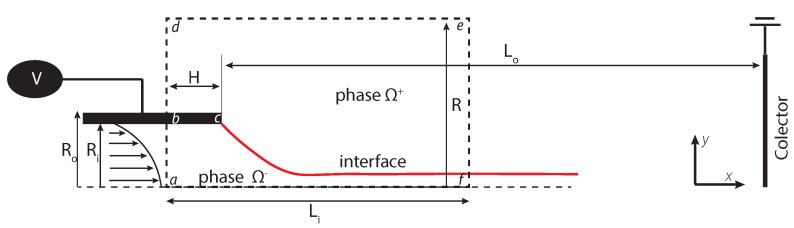
A schematic of the physical domain. The rectangle represented by dashed lines indicates the computational domain.

The physical domain is shown in Figure 5. In the same figure, dashed rectangle shows the axisymmetric domain used in the numerical simulation. The overall domain is decomposed into two main parts: the outer region $\Omega^{+}$*(gas)* and the inner region $\Omega^{-}$ *(liquid)*, separated by the interface.

The inner phase represents the polymer solution (represented by subscript i), located inside the needle outer phase (represented by subscript o) is the surrounding gas.

The dimensions of the emitter are as follows: inner radius $R_{i}=0.2$ mm, outer diameter $R_{o}=0.25$ mm, emitter length H=0.5 mm. The distance $L_{o}$ between the emitter and the extractor electrode is 120 mm, and the surrounding area is, in principle, infinite. The region in the vicinity of the emitter (Figure 5) is the one where the EHDA phenomenon takes place. A small simulation domain is used to avoid computational overhead. The dimension of the small domain is: the distance $L_{i}$ is about $15R_{i}$, the distance R=5 mm. Similar domains are used in the study of Xu et al. [29]. Complementary, boundary conditions established to perform the simulation are summarized in Table 2.

### 4.2. Validation Case: Taylor Cone Formation

Electrospray technique works for a range of voltages and flow rates, not limited to the ones characterized by Taylor cone formation [48]. However, as already mentioned above, Taylor cone characterizes the configuration that results in droplets of uniform size, which is favorable for practical purposes. Thus, we concentrate here on the Taylor cone formation. The perfect cone shape (Taylor cone) feature is not universal, the shape depends on flow rate and solution properties [49]. As mentioned in the Introduction, in the original work of Taylor, the angle of 49.3° was reported for the solution composed of water mixed with oil. Yarin et al. [50] has reported that for aqueous solution of polyethylene oxide (PEO, K400) the half-angle of the cone shape is 30.5°. Afterwards, Michelson [51] suggested that the stable cone from low-viscosity liquids is close to 45°. Considering that for the analyzed PVP-DMF solutions, the Taylor cone half-angle was not known a priori, the formation of the stable cone was analyzed experimentally.

**Figure 6:**
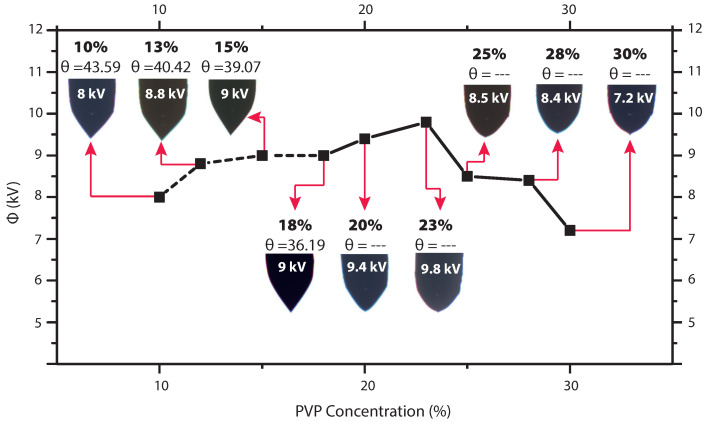
The photographs showing the effect of PVP concentration and voltage on the shape and half-angle of the Taylor cone.

To produce a conical meniscus (Taylor cone), the flow rate (Q) and the distance to the collector were set to 0.1 mL/h and 12 cm, respectively. The voltage was varied from 7.2 to 10 kV to achieve a cone shape. The snapshots of the formation of the meniscus were taken using a CCD camera, and are shown in Figure 6.

**Figure 7:**
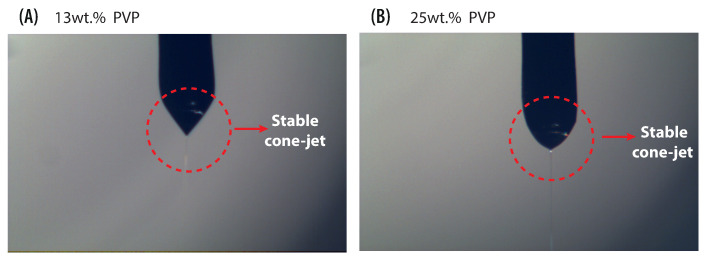
Images of cone-jet formation for PVP concentration of 13 wt% and 25 wt%. The red circle shows the cone shape, the jet emerging from the tip of the cone.

One can see that the Taylor cone was obtained for concentrations of 10%, 13%, 15%, and 18% (discontinuous line). Corresponding figures exhibit a well-defined conical shape. The concentration of about 20% can be considered a transition point, where the cone begins to change to a smoothed tip shape. The voltages at which the stable cone shape formed are also displayed in Figure 6. The angle measurements for the last four concentrations were not taken, as in these cases the conical shape is completely lost. For concentrations above 20%, it was observed that droplet emission still took place. Droplets and jet increase in size when the PVP concentration increases to 25 wt%, it was also noted that the shape of the cone changed from a sharp tip to a rounder tip. (Figure 7A,B), the jet becomes thicker and larger similar observations were reported [8,52].

The results of the experiments performed highlight that the value of the half-angle decreases as the polymer concentration increases. According to Yarin et al. [50], the acuteness of the cone depends on viscosity, elasticity, and surface tension of the solution. One can see that the cone angle decreases from 43.6° (in case of 10% solution) to around 36° (when concentration reaches 18%). Overall, the obtained half-angle value for solutions with low viscosity matches with Michelson [51] and confirms the findings of Yarin et al. [50]. As anticipated, Taylor cones never show static features [48]. During the experiments, it was possible to observe an instantaneous perturbation which tended to reduce the cone angle. This anomaly may be attributed to reduced electrostatic shielding and was reported also in [49]. In our case, the 13 wt% and 25 wt% concentrations presented the most stable operation regime, for this reason, these settings were chosen to perform the numerical simulation.

Different ingredients of the numerical solver described in Section 2 have already been validated in various examples. The electrostatic solver was validated in Roghair [53]. Validation of the two-phase flow solver in application to microfluidic problems was performed, among others, by Deshpande et al. [54]. Mentioned work confirmed that the solver was able to capture the physics associated with the Rayleigh breakup of a laminar jet. Narváez-Muñoz validated the electromechanical coupling by comparing the results from numerical simulation of electrosprayed heptane droplets with experimental data [55], the size of the droplets was in good agreement with the experimental data of Gomez and Tang [56].

**Figure 8:**
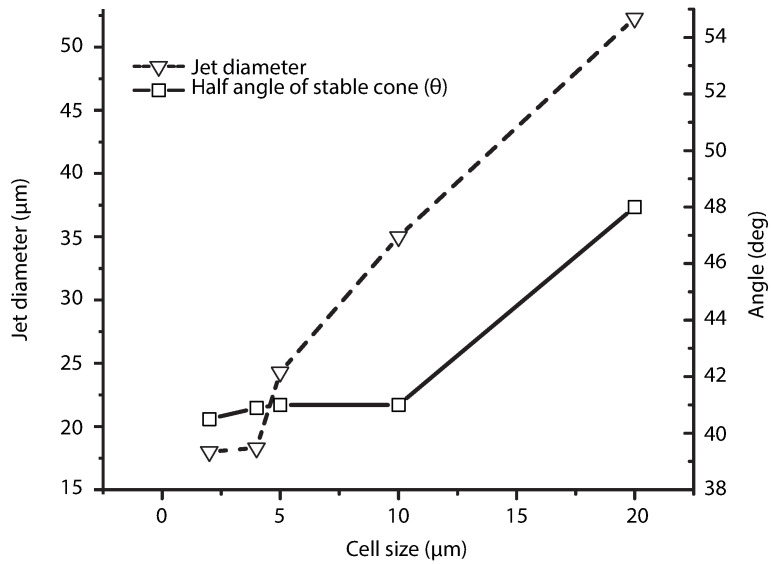
Plot of jet diameter and half-angle versus minimum cell size for 13 wt% PVP solution.

Below we test the ability of the electrohydrodynamics solver to reproduce the Taylor cone in the problem at hand. This validation is performed by comparing the images obtained in the experiment with the results of the numerical simulation. The flow rate and the voltages leading to stable cone formation (according to the experiment) are applied as the boundary conditions in the numerical simulation, which analyzes the evolution of the meniscus. Flow rate 0.1 mL/h and electric field 0.66 kV/cm were used to perform the numerical study for a PVP solution at 13 wt% concentration. The flow rate value is close to the scaling law prediction for the minimum value for which a stable cone-jet is expected to be reached $Q_{o}\approx 0.01$ mL/h ($Q_{o}\approx \gamma \epsilon_{0}\epsilon /\rho \sigma$) [57]. For the numerical simulation, the *blockMesh* utility of OpenFOAM was used to discretize the domain with structured non-uniform grid, the coarse mesh is on the sides while finest one is located at the needle. Six different mesh sizes were tested to examine convergence. The following minimum cell size 1, 2, 3, 5, 10, and 20 $\times \;10^{-6}$ m were used. Figure 8 illustrates the jet diameter and the half-angle at different mesh resolutions. One can see that for grid size of ≤2 $\times \;10^{-6}$ m convergence is reached. This resolution ($2\times 10^{-6}$ m) is used in all the tests described below unless specified otherwise. It results in a mesh of approximately 450,000 cells.

**Figure 9:**
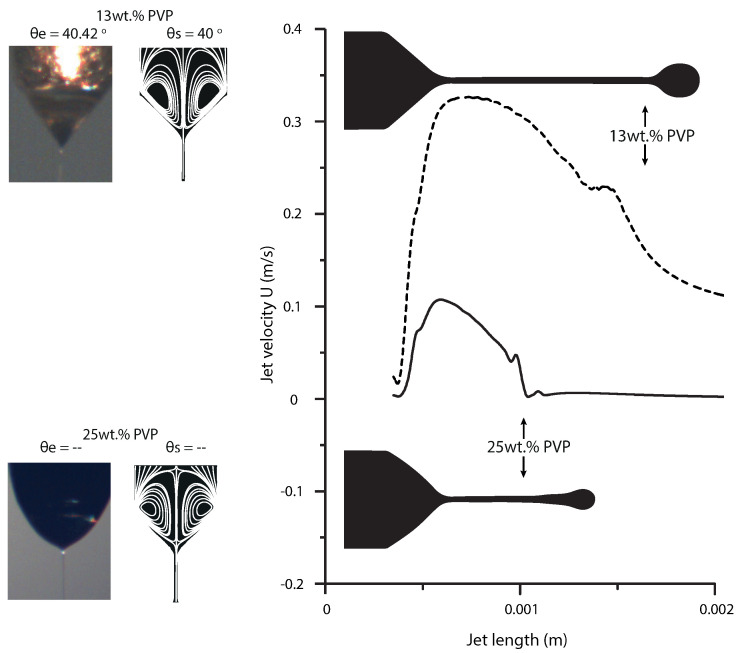
Mean flow velocity along the longitudinal axis at 60 ms for PVP solutions of 13% (Q = 0.1 mL/h, ϕ = 8 kV, μ = 9.4 mPa.s) and 25% (Q = 0.1 mL/h, ϕ = 7.7 kV, μ = 38.5 mPa.s). Contours with streamlines plotted and experimental photographs, showing the effect of PVP concentration on the shape and half-angle of the Taylor cone.

In what follows we examine the meniscus for two different PVP concentrations. Figure 9 shows the numerical simulation results obtained for 13 wt% and 25 wt% of PVP. One can see that the increase of polymer concentration significantly affects the conical shape and velocity of the PVP solution. This agrees well with the experimental finding and previous studies [12,58,59]. Additionally, to the evolution of meniscus shape we present the velocity plot along the longitudinal axis at 60 ms for both concentrations of PVP (see Figure 9). Please note that the velocity of the solution in the jet is higher compared to its velocity in the meniscus [60]. The characteristic velocity inside the Taylor cone can be estimating by $U\approx \left(\epsilon_{0}\gamma I^{2}\right)/{\left(\rho \mu \sigma^{2}L_{c}^{4}\right)}^{1/3}$, where *I* is the current ($I\approx {\left(\gamma \sigma Q\right)}^{1/2})$ and L${}_{c}$ is the characteristic meniscus length (diameter of the needle used) [61]. The above data yields U ≈ 5.1 × 10${}^{-4}$ and U ≈ 2.8 × 10${}^{-4}$ m/s for PVP solutions at 13% and 25%, respectively. It was found in the numerical simulation for the same concentrations that the axial velocity inside the meniscus is close to this characteristic velocity, U${}_{x}$ ≈ 4.1 × 10${}^{-4}$ and U${}_{x}$ ≈ 3.6 × 10${}^{-4}$ m/s, respectively. On the other hand, the figure shows that the cone-jet length decreases while viscosity increases. As the velocity distribution shows, the cone-jet occurs later in solutions with higher viscosity.

Ultimately, we compare the stable shape obtained numerically with the one observed in experiments. The comparison for both concentrations considered is shown in Figure 9. The numerical results are juxtaposed with the experimental one. One can see that the numerical contours adequately capture the change of the stable shape. In the case of 13% PVP concentration Taylor cone is clearly observed, the deviation between the tip angle obtained experimentally ($\theta_{e}$) and numerically ($\theta_{s}$) is less than 1°. For the solution of 25% cone shape is not formed, thus the angle was not measured. However, one can see that the stable shape obtained numerically is very similar to the one recorded in the experiment. Overall, one can conclude that the results of our electromechanical numerical model employed in this work are consistent with the experimental evidence which indicates that the model is suitable for the simulation of the phenomenon under consideration.

### 4.3. Prediction of the Operational Parameters

The flow rate and the applied voltage are the key parameters to define a finite island of stability in an EHDA process [62]. These two parameters can vary within a limited range, beyond which the Taylor cone is no longer stable [63]. The stability lower bound is defined by the minimum flow rate and its associated voltage, which have been identified experimentally in the previous section for various PVP solutions. Previous studies reported scaling relation to calculate the minimum voltage (Equation (18)) to form a steady cone-jet [58,64,65,66,67]. However, such approach does not account for the influence of the flow rate and liquid viscosity. Although these parameters affect the kinetic energy and the charge density on the cone-jet, they are not taken into consideration in the onset voltage.

(18) $$\phi_{o}=\sqrt{\frac{\gamma d_{o}}{2\epsilon_{0}}}ln\left(\frac{4L_{0}}{d_{o}}\right)$$

**Figure 10:**
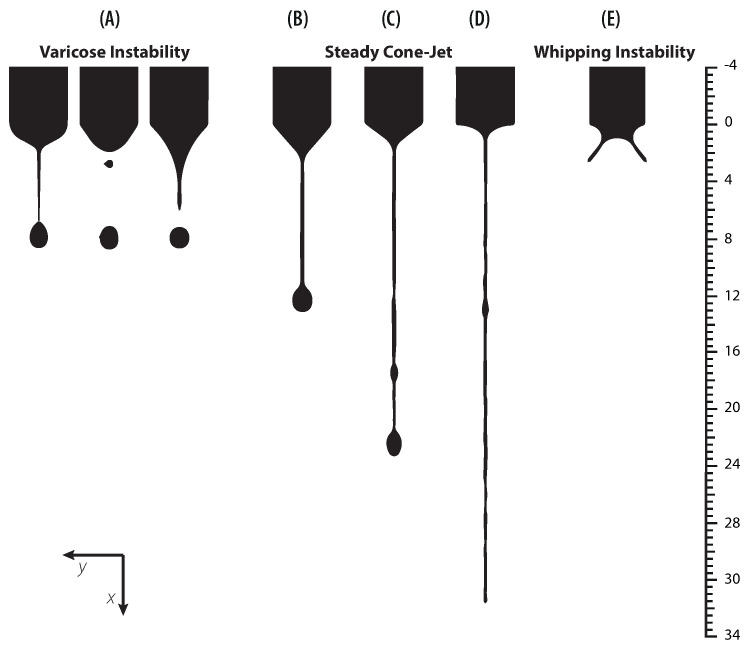
Profile of the cone-jet formation for a concentration of 13% PVP and the major ticks mark represents 400 μm. (**A**) Q = 0.1 mL/h V = 4 kV at t = 25,35,45 ms, (**B**) Q = 0.1 mL/h V = 8 kV at t = 60 ms, (**C**) Q = 0.6 mL/h V = 18 kV at t = 60 ms, (**D**) Q = 0.6 mL/h V = 19.5 kV at t = 60 ms, and (**E**) Q = 0.6 mL/h V = 23 kV at t = 5 ms.

**Figure 11:**
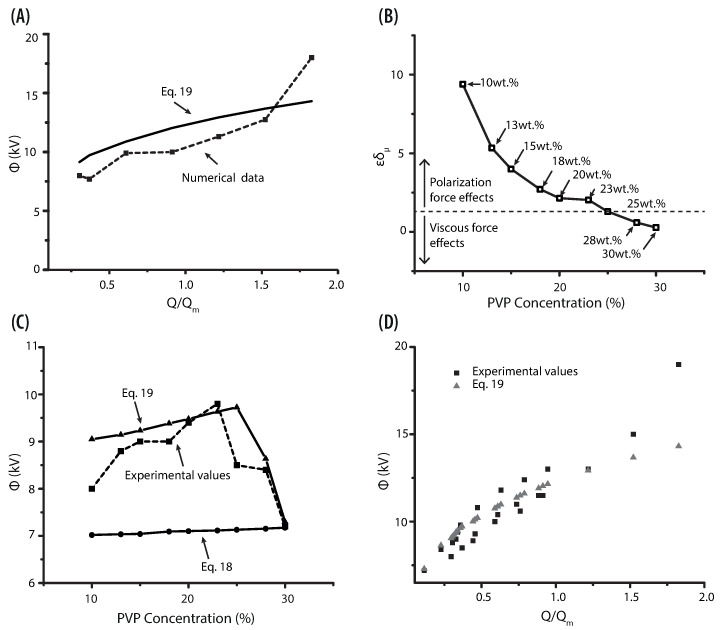
(**A**) The scaling relationship for 13 wt% and 25 wt% PVP at different flow rates. The dotted line represents the operational parameters from the numerical simulation. (**B**) Electrohydrodynamic Reynolds number versus PVP concentration. (**C**) Plot of the voltage as function of PVP concentrations. The dotted line corresponds to the experimental values for stable cone-jet, while the solid lines represents the prediction using Equations (18) and (19). (**D**) Voltage as a function of dimensionless flow rate for PVP at different concentration.

Below we use the computational model, modifying the flow rate, voltage and polymer concentration to determine the upper bound of the stability island, i.e., the operating parameters will be altered to identify the threshold for the onset of the cone shape instability. The onset of the multi-jet mode can be considered the indication of reaching the upper limit of the stability island, at this point, the cone starts to skew whipping instability (Figure 10). One can see that an increase in the flow rate results in the cone stretching in addition to the thickening of the cone-jet radius. The surface tension weakens, and the hydrodynamic pressure intensifies as flow rate increase [34]. These observations provide the guidelines for determining the stable zone. In the current study, the flow rate and voltage were increased up to 0.6 mL/h and 18 kV, respectively. Figure 11A shows the numerical prediction (square markers), as a function of the voltage and dimensionless flow rate ($Q/Q_{m}$). Two regimes were identified when the flow rate approached $Q_{m}$ (Figure 11B). When the polarization force destabilizes the cone-jet, $\epsilon \delta_{\mu }>1$, where $\delta_{\mu }={\left[\gamma^{2}\rho \epsilon_{0}/\left(\mu^{3}\sigma \right)\right]}^{1/3}$ is the electrohydrodynamic Reynolds number, then $Q_{m}=\epsilon Q_{o}$. On the other hand, when the viscous force destabilizes the cone-jet $\epsilon \delta_{\mu }<1$, in this case $Q_{m}$ scales with $Q_{m}=Q_{o}/\delta_{\mu }$ [9]. The data in Figure 11B shows that the solution at 25 wt% is where the viscous forces opposing the liquid motion. This finding supports the velocity difference of the more viscous solution, which was reported in the above section.

The results of the correlational analysis (solid line) are set out in the aforementioned figure (Figure 11A). Considering it, the starting voltage for an electrospray process, can be defined by the equation:

(19) $$\phi =A\sqrt{\frac{\gamma d_{o}}{2\epsilon_{0}}}ln\left(\frac{4L_{o}}{d_{o}}\right){\left(\frac{Q}{Q_{o}}\right)}^{0.25}$$

where the empirical coefficient *A* is 1.75. From the relation proposed, we could predict the onset voltage required for the formation of a stable cone-jet in different PVP solutions. A comparison between experimental values and equations used to calculate the onset voltage is shown in Figure 11C. The solid lines represent the values of the Equations (18) and (19). What stands out in the mentioned figure is that Equation (19) correlates the response of the solution parameters (viscosity) and operational parameters (flow rate). Including such parameters gives a more accurate value of the onset voltage than those reported by Equation (18). A plot of the experimental values of Q and ϕ for all PVP concentrations is shown in Figure 11D. The triangles represent Equation (19), while the squares indicate the experimental values. Although the results follow the same trend, they show a bare difference when the flow rate reaches the highest values. However, most of the applications operate at low flow rates as at high flow rates a regime with two populations of particles (main and satellite) evolves [20,57].

For determining the stability island, the flow rate was increased in steps of 0.1 mL/h until reaching the limit, while the corresponding voltages were found when the cone shape showed similar features to those observed in the experiments. At values of the lower bound (Q = 0.1 mL/h; V = 8 kV) the meniscus forms a convex conical shape, and it transforms to a concave one once the upper limit is reached (Q = 0.6 mL/h; V = 18 kV). Despite increasing the voltage to compensate for the rising velocity, the motion of the fluid is driven by the hydrodynamic pressure and the expansion of the jet diameter and cone is inevitable. Similarly, at low values of flow rate the length of the jet is smaller. Eventually, the droplets are formed at the end of the jet, as the concentration of the polarization force and the surface tension tends to restore the equilibrium on the jet, it decreases and increases in diameter until breakup. Similar behavior was reported by Hohman et al. [68].

**Figure 12:**
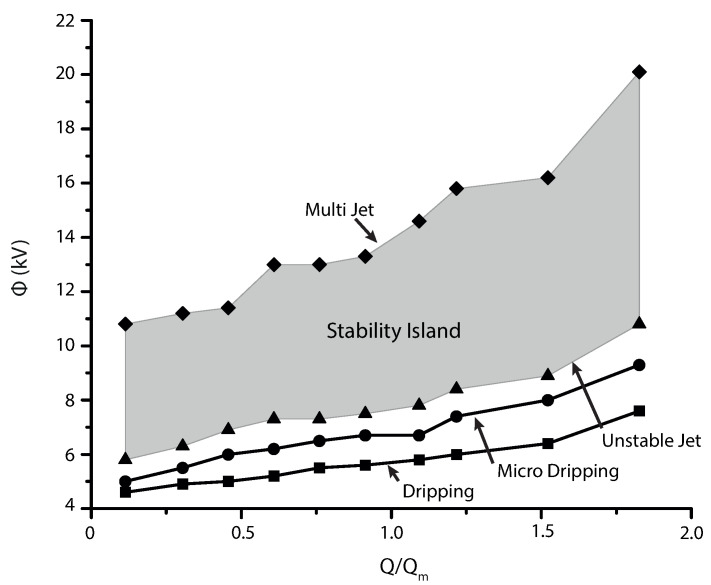
Prediction of the stability island; Above of the island, multi-jet is produced due to the high electric field; Below the stability region, stable cone-jet is not periodic.

Considering the above, one can confirm that operating parameters may be affected by the viscosity. Flow rate and voltage play an important role in controlling the stability of the cone. Moreover, the collected data allowed determination of the correlation between these variables, which describes the stability region (Figure 12). According to the performed analysis, stability island corresponds to the gray region denoting stable jet, where dimensionless flow rate and voltage are represented by X and Y axes, respectively. If the operational point is located at the predicted interval the polymer solution may be successfully electrosprayed, regardless of the particle quality. This observation means that the electrospray process is stable for a wide range of applied voltages and flow rates, and the proposed relationship can be used as a simple means for predicting the operation parameters.

## 5. Fabrication of PVP Structures

**Figure 13:**
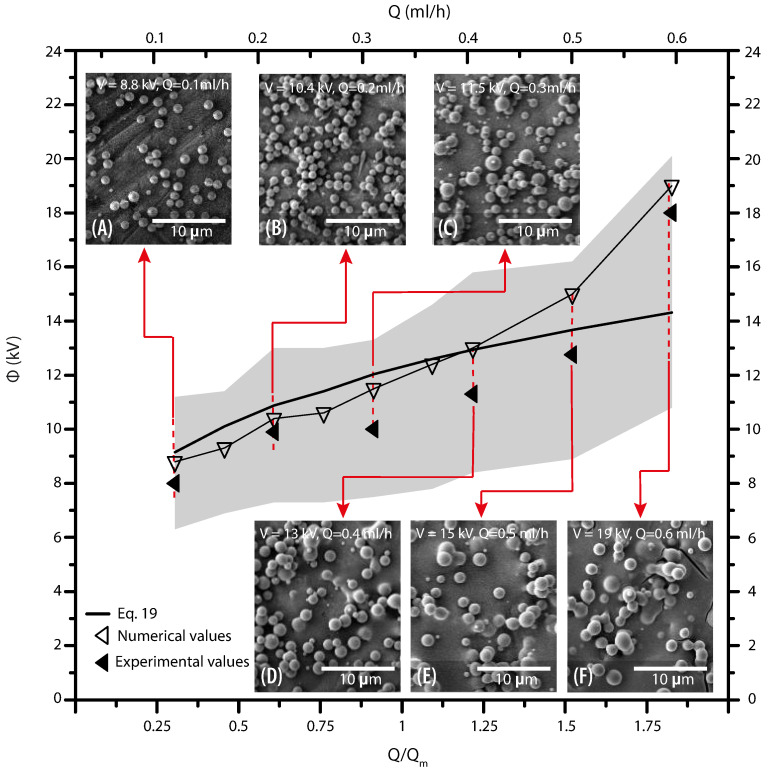
Operating diagram to fabricate particles from 13 wt% PVP solution. SEM images of the particles as insets for each numerical prediction. Particles diameter $d_{p}$ for; (**A**) Q = 0.1 mL/h V = 8.8 kV ($d_{p}\approx 1.17\pm 0.13$ μm), (**B**) Q = 0.2 mL/h V = 10.4 kV ($d_{p}\approx 1.23\pm 0.5$ μm), (**C**) Q = 0.3 mL/h V = 11.25 kV ($d_{p}\approx 1.28\pm 0.5$ μm), (**D**) Q = 0.4 mL/h V = 13 kV ($d_{p}\approx 1.39\pm 0.24$ μm), (**E**) Q = 0.5 mL/h V = 15 kV ($d_{p}\approx 1.51\pm 0.27$ μm), and (**F**) Q = 0.6 mL/h V = 19 kV ($d_{p}\approx 1.7\pm 0.27$ μm).

**Figure 14:**
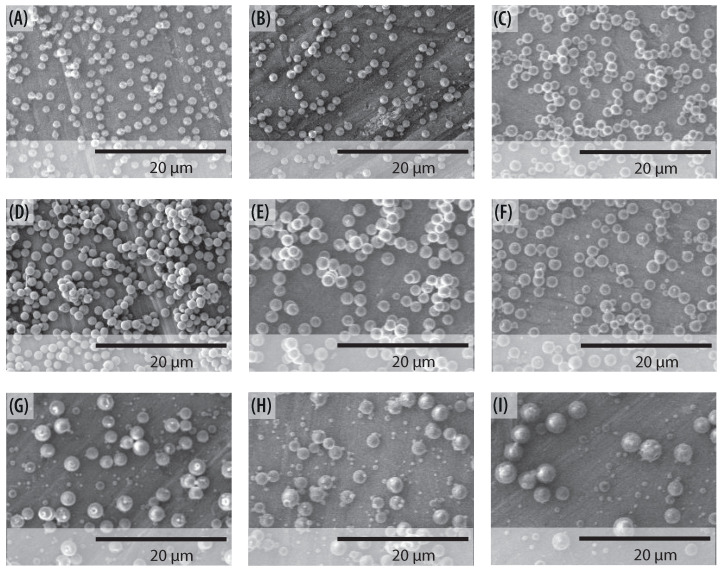
SEM images of fabricated particles from different PVP concentrations at Q = 0.1 mL/h. Particles diameter $d_{p}$ for; (**A**) 10 wt% PVP at V = 8 kV ($d_{p}\approx 1.08\pm 0.15$ μm), (**B**) 13 wt% PVP at V = 8.8 kV ($d_{p}\approx 1.17\pm 0.14$ μm), (**C**) 15 wt% PVP at V = 9 kV ($d_{p}\approx 1.28\pm 0.36$ μm), (**D**) 18 wt% PVP at V = 9 kV ($d_{p}\approx 1.35\pm 0.49$ μm), (**E**) 20 wt% PVP at V = 9.4 kV ($d_{p}\approx 1.71\pm 0.49$ μm), (**F**) 23 wt% PVP at V = 9.8 kV ($d_{p}\approx 1.77\pm 0.17$ μm), (**G**) 25 wt% PVP at V = 8.5 kV ($d_{p}\approx 1.85\pm 0.34$ μm), (**H**) 28 wt% PVP at V = 8.4 kV ($d_{p}\approx 1.9\pm 0.34$ μm), and (**I**) 30 wt% PVP at V = 7.2 kV ($d_{p}\approx 2.11\pm 0.71$ μm).

In the present section, we describe how the stability island established in Section 4.3 can be used to set up the operational parameters. Figure 13 shows the SEM images of produced particles using various operation parameters. The collecting distance was set to 12 cm. The predicted values of voltage are taken as a starting point, the onset values were fitted until achieving the stable cone-jet mode. No significant differences were found between the predicted and the set values, while, as expected, the value of the operational parameters are within the region of the stability island. The carried out electrospraying produced particles whose average diameter was varied by changing the flow rate. For all the cases the particles present a spherical shape. However, when flow rates range from 0.4 mL/h to 0.6 mL/h the solvent was not completely evaporated, even when the voltage has increased. Observing Figure 13, one can clearly conclude that smooth and uniform particles were obtained in the lower region of the island. It is noted furthermore that the diameter of the particle depends on the flow rate and the voltage as suggested in the literature. [57]. Figure 14 shows the SEM photographs of the electrosprayed particles made for all the concentrations of PVP, with a constant flow rate equal to 0.1 mL/h, a collecting distance of 12 cm, and different voltage for each concentration. For all the concentrations the particles present a spherical shape, which indicates fully solvent evaporation. However, for larger content of PVP in the solution, one obtains a less homogeneous distribution of particle diameter. Figure 14F,I) shows that despite the overall spherical shape of the particles, there are surfaces with some irregularities. This occurs because, at higher concentrations, satellite droplets are generated.

**Figure 15:**
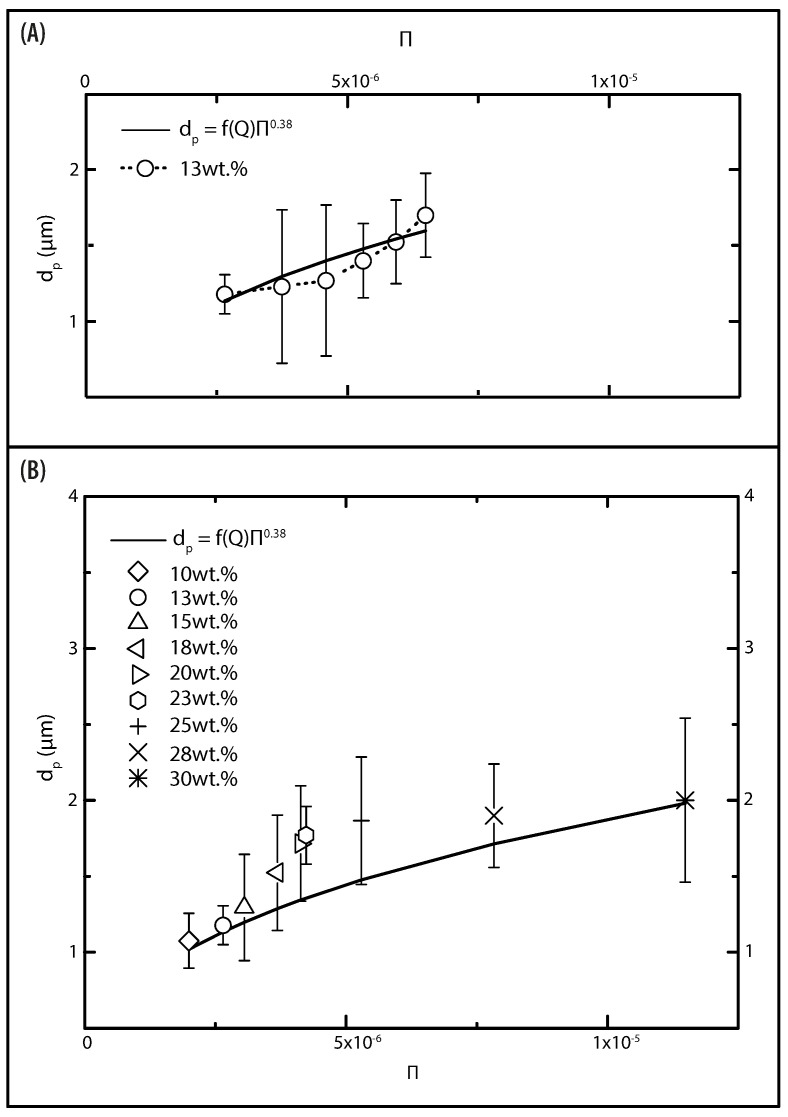
Plot of the particle diameter $d_{p}$ as function of Π. The solid line represents the scaling relationship. (**A**) Electrosprayed particles from 13 wt% PVP solution at different flow rates. (**B**) Particles fabricated from all PVP concentrations, flow rate remained constant (Q = 0.1 mL/h).

To analyze the particle size dependency, the experimental data Q, μ, and γ are merged into the dimensionless number $\Pi =\sqrt{\mu Q/\gamma }$. The flow rate dependency on the particle size was analyzed using the 13 wt% PVP solution (Figure 15A). When the flow rate decreases to the minimum value (Q = 0.1 mL/h), the particles have a smaller diameter ($d_{p}$ = 1.17 μm) and possess a smooth surface and narrow particle size distribution. On the other hand, for high flow rate (Q = 0.6 mL/h) larger particles were obtained ($d_{p}$ = 1.7 μm). The data shows a clear tendency: the particle diameter increases as the flow rate increases. Flow rate-particle diameter follows a linear relation:

(20) $$d_{p}=f\left(Q\right){\left(\sqrt{\mu Q/\gamma }\right)}^{0.38}$$

The value of the conversion factor (f(Q)) is 149.38. Despite the flow rate dependency, it is reported in the literature that particle size strongly depends on the viscosity of the solution [21]. Particle diameter as a function of viscosity and surface tension is plotted in Figure 15B. There is a clear trend towards an increase in the $d_{p}$ as the μ/γ increased. One must note that the discrepancy between the predicted diameter and experimental results may be attributed to various factors, such as temperature and humidity. Such conditions may lead to variations in the electric potential distribution, which affect particle formation [69]. Despite this limitation, our results capture the influence of the operational parameters and solution properties on the formation of particles. Hence, the power scaling (Equation (20)) may be used to predict the diameter of the electrosprayed particles. The other result, which can be considered of particular importance, is the one that emerged from the data of the morphological irregularities. It suggests that such irregularities are controlled by the flow rate, while the particle diameter is controlled by the viscosity of the solution.

## 6. Summary and Conclusions

The influence of electrospray parameters, such as flow rate, electric field, and polymer concentration, has been investigated using a combination of experimental and numerical approaches for solutions of PVP in DMF. Performed study provided evidence that the half-angle of the stable cone (Taylor Cone) and the corresponding operational parameters are affected by the viscosity of the polymer solution. The scaling law for the onset voltage was obtained by considering the nature of the force opposite to the motion of the PVP solution. Interestingly, when the viscous force destabilizes the cone-jet the onset voltage decreases, while when the polarization force generates the instabilities, the voltage increases.

As a result of the present study the stability island for producing spherical PVP particles was identified. The results for lower flow rates showed that the particles obtain a perfectly spherical shape, while for high flow rates the solvent is not fully evaporated, demonstrating the waste of material. This suggests that the flow rate significantly impacts shape irregularities during the electrospray process. The size of the spherical particles is impacted by the viscosity of the solution. At high PVP concentrations, satellite particles appear. It was determined that the viscosity and flow rate influence the size of the particles and can be well approximated by the scaling law $d_{p}=f\left(Q\right)\Pi^{0.38}$. Overall, the results of this study provide the basis for the production of monodisperse microspheres through electrospray technique which may be used for various applications, such as drug delivery, bio-imaging, photocatalytic, sensing, among others.

The use of the combined numerico-experimental approach has proven to be effective for finding optimal operational parameters for PVP particles fabrication. Such a combination considerably reduces the analysis cost of electrospray process in comparison with the purely experimental methods.

## Figures and Tables

**Table 1:** Properties of the PVP-DMF solutions. All the measurements were performed at room conditions.

PVP wt%	σ (μS/cm)	γ (mN/m)	μ (mPa.s)	ρ (kg/m${}^{3}$)
10	52.4	36.99	5.3	965
13	52.6	37.17	9.4	990
15	54	37.24	12.51	1001
18	56	37.77	18.48	1015
20	57.4	37.88	23.3	1028
23	59.4	38.02	24.6	1056
25	60.8	38.18	38.5	1067
28	62.1	38.41	84.8	1092
30	63	38.65	183.6	1158

**Table 2:** Boundary conditions used in the computational domain (note that Fixedfluxpressure b.c. of the OpenFoam sets the pressure gradient to the provided value such that the flux on the boundary is that specified by the velocity boundary condition).

Boundary	U	p	ϕ	$\rho_{e}$
a–b	U = fixed value	Fixedfluxpressure	N/a	∇$\rho_{e}$ = 0
b–c	U = 0	Fixedfluxpressure	ϕ	∇$\rho_{e}$ = 0
b–d	zeroGradient	p = 0	N/a	∇$\rho_{e}$ = 0
d–e	zeroGradient	p = 0	N/a	∇$\rho_{e}$ = 0
e–f	zeroGradient	Fixedfluxpressure	0	∇$\rho_{e}$ = 0
f–a	zeroGradient	Fixedfluxpressure	N/a	∇$\rho_{e}$ = 0

## Data Availability

The data presented in this study are available on request from the corresponding author.
